# SECTM1 is upregulated in immuno-hot tumors and predicts immunotherapeutic efficacy in multiple cancers

**DOI:** 10.1016/j.isci.2023.106027

**Published:** 2023-01-23

**Authors:** Jie Mei, Ziyi Fu, Yun Cai, Chenghu Song, Jiaofeng Zhou, Yichao Zhu, Wenjun Mao, Junying Xu, Yongmei Yin

**Affiliations:** 1 Department of Oncology, The Affiliated Wuxi People’s Hospital of Nanjing Medical University, No. 299 Qingyang Road, Wuxi 214023, China; 2 Department of Oncology, The First Affiliated Hospital of Nanjing Medical University, No. 300 Guangzhou Road, Nanjing 210029, China; 3 Wuxi Clinical College of Nanjing Medical University, No. 299 Qingyang Road, Wuxi 214023, China; 4 Department of Thoracic Surgery, Department of Cardiothoracic Surgery, The Affiliated Wuxi People’s Hospital of Nanjing Medical University, No. 299 Qingyang Road, Wuxi 214023, China; 5 Department of Physiology, Nanjing Medical University, No. 818 Tianyuan East Road, Nanjing 211166, China; 6 Jiangsu Key Lab of Cancer Biomarkers, Prevention and Treatment, Collaborative Innovation Center for Personalized Cancer Medicine, Nanjing Medical University, No. 818 Tianyuan East Road, Nanjing 211166, China

**Keywords:** Biological sciences, Bioinformatics, Bioinformatic numerical analysis, Genomics

## Abstract

Immune checkpoint inhibitors (ICIs) have transformed the management of advanced cancers. However, many patients could not benefit from ICIs therapy, and thus several biomarkers for therapeutic prediction have been uncovered. In this research, more than ten public and in-house cohorts were used to explore the predictive value and immunological correlations of secreted and transmembrane 1 (SECTM1) in cancers. SECTM1 expression was enhanced in tumors from patients with well immunotherapeutic responses in multiple cancers. In addition, SECTM1 was immuno-correlated in pan-cancer and enhanced in immuno-hot tumors. *In vitro* assays revealed that SECTM1 was upregulated by the IFN-γ/STAT1 signaling. Moreover, analysis of in-house immunotherapy cohorts suggested both tumor-expressed and circulating SECTM1 are promising biomarkers to predict therapeutic responses. Overall, this study reveals that SECTM1 is a biomarker of benefit to ICIs in cancer patients. Further studies including large-scale patients are needed to establish its utilization as a biomarker of benefit to ICIs.

## Highlights

• SECTM1 predicts the responses to immunotherapy in multiple cancer types
• SECTM1 is associated with tumor immune microenvironment across cancer types
• SECTM1 is upregulated by IFN-γ/STAT1 signaling in immuno-hot tumors
• Tumor-expressed and circulating SECTM1 predict immunotherapeutic responses

## Graphical abstract

**Figure:**
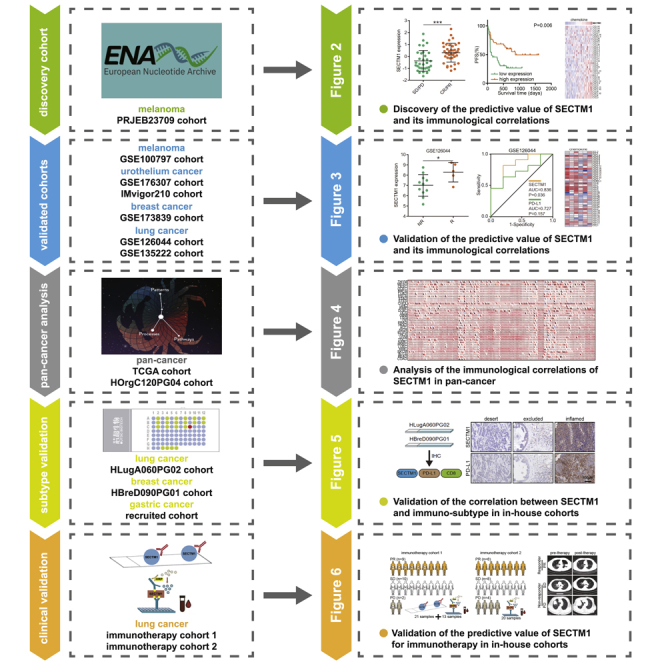

## Introduction

In the past decades, immunotherapy has been a revolutionary strategy and has largely transformed the therapeutic situation of human cancers with advanced clinical stages.^1^ Immunotherapy aims to activate the patient’s immune system, which relies on the immune function to kill tumor cells, and the most widely used immunotherapy is to block the interplay between immune checkpoints expressed on malignant and anti-tumor immune cells using immune checkpoint inhibitors (ICIs).^2^ Programmed death-ligand 1 (PD-L1) is an immunosuppressive immune checkpoint mainly expressed on tumor cells^3^ and plays a crucial role in triggering tumor cell immune escape by binding to its receptor programmed cell death protein 1 (PD-1) expressed on anti-tumor immune cells.^3^^,^^4^ Although the prognosis of cancer patients with advanced stages has been persistently improved with the application of immunotherapy, not all patients could benefit from the established treatment options.^5^^,^^6^ It has been well known that PD-L1 expression is a dominating factor that determines whether a patient responds to anti-PD-1/PD-L1 immunotherapy, but a large group of patients with PD-L1-negative expression could also benefit from immunotherapy.^7^^,^^8^ Thus, complementary and alternative biomarkers are urgent in clinical practice for the prediction of immunotherapeutic responses.

Secreted and transmembrane 1 (SECTM1) is a transmembrane and secreted protein with features of a type 1a transmembrane protein.^9^ SECTM1 is expressed on cytomembrane, which is identified as a CD7 ligand.^10^^,^^11^ CD7 is expressed in T and natural killer (NK) cells,^11^ which could be activated by its ligand SECTM1, thus promoting the proliferation of T and NK cells.^12^ It has been found that SECTM1 strongly co-stimulates CD4^+^ and CD8^+^ T cell proliferation and promotes the production of interferon gamma (IFN-γ) in a CD7-dependent manner.^12^ Based on its cellular functions, SECTM1 is significant for hematopoietic and immune system processes. It has been revealed that SECTM1 is overexpressed in melanoma tissues.^13^ However, the functional role of SECTM1 in human cancers and its correlations with anti-tumor immunity have not been investigated.

In this research, we aimed to explore the immunological correlations of SECTM1 in human cancer. We utilized a panel of public datasets to explore the predictive value of SECTM1 for the responses to immunotherapy and its immunological correlations. In addition, the immunological correlations of SECTM1 in pan-cancer were systematically analyzed using The Cancer Genome Atlas (TCGA) dataset and validated using four independent in-house cohorts. Moreover, the predictive value of SECTM1 for the responses to immunotherapy was also validated using two independent clinical cohorts. Overall, we illustrated the significant function of SECTM1 in mediating anti-tumor immunity and identified SECTM1 as a novel and promising biomarker for the prediction of immunotherapeutic responses in multiple cancers.

## Results

### The overall design of the current study

**Figure 1:**
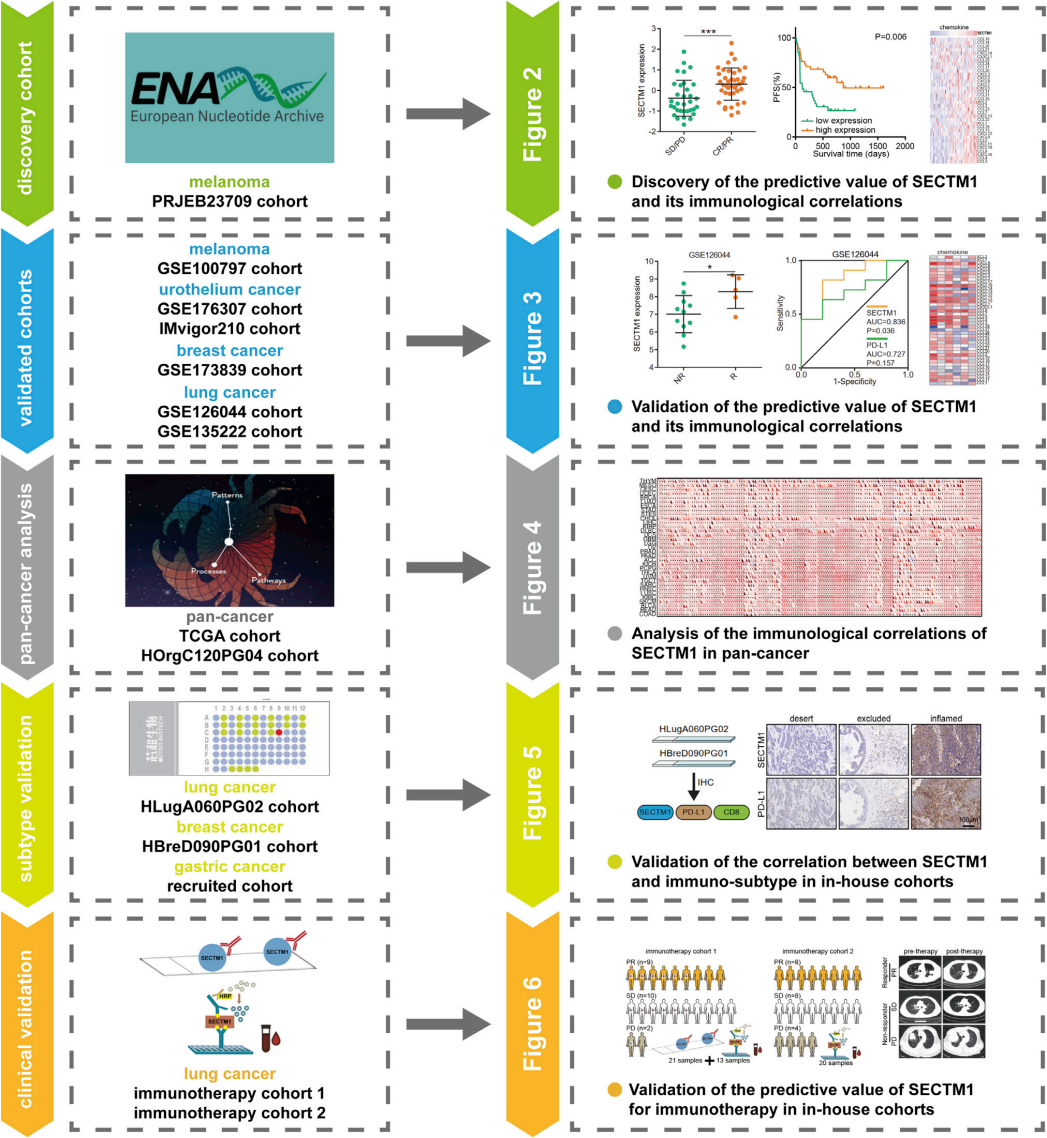
The flow chart of the current study The current research explored the predictive value and immunological correlations of SECTM1 in cancers using a panel of public cohorts and in-house cohorts and finally identified SECTM1 as a novel biomarker for the prediction of immunotherapeutic responses.

The current research aimed to check the predictive value and immunological correlations of SECTM1 in human cancer. The predictive value and immunological correlations of SECTM1 were first discovered in the PRJEB23709 cohort and then were validated using six independent cohorts. Next, the immunological correlations of SECTM1 in pan-cancer were explored using the TCGA dataset. Furthermore, we also evaluated the expression of SECTM1 in tumors with different immuno-subtypes, its association with mismatch repair (MMR) status, and its predictive value using multiple independent in-house cohorts. The overall design of the current study was shown in Figure 1.

### SECTM1 is a promising biomarker of benefit to immunotherapy in melanoma

**Figure 2:**
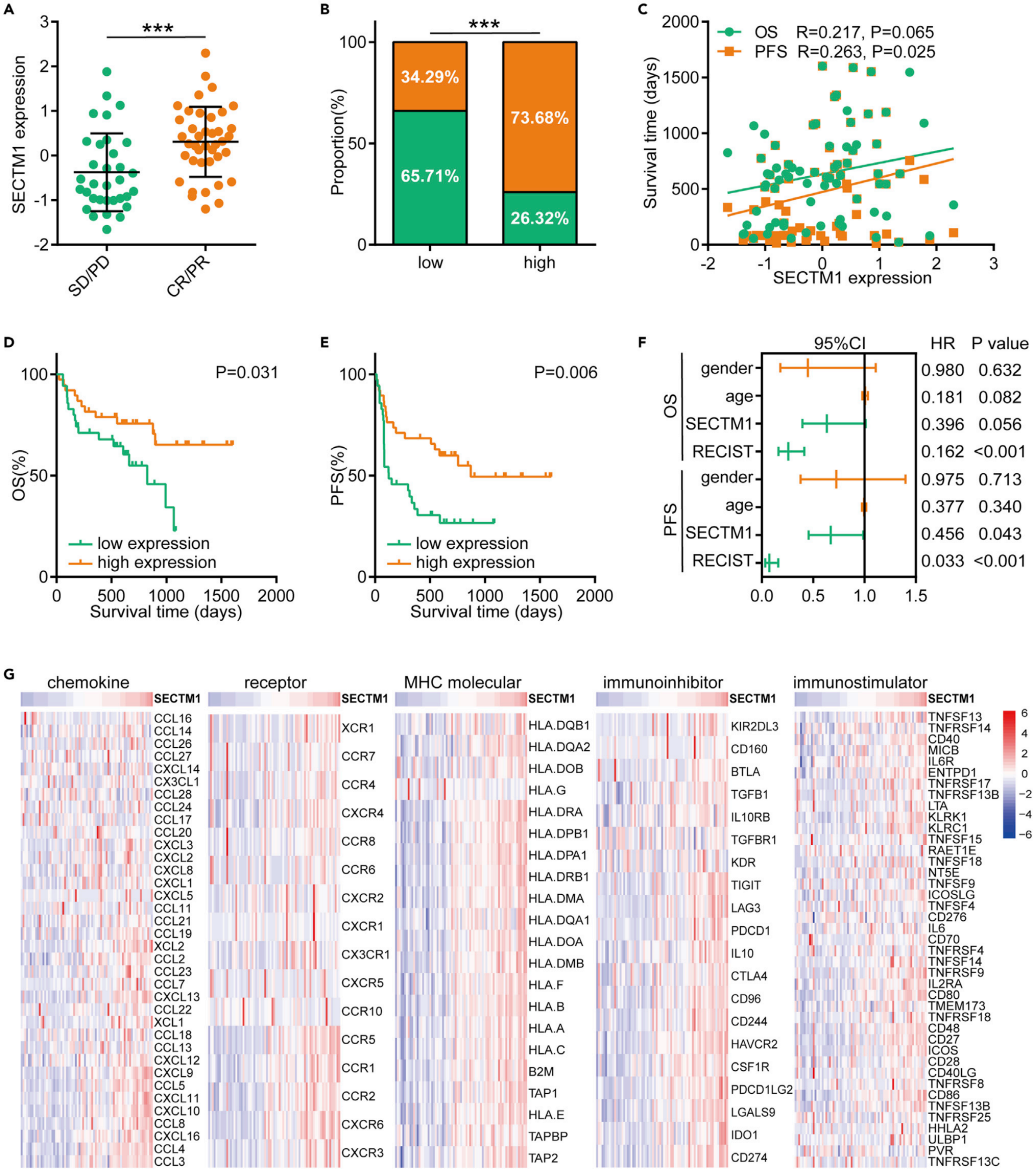
Predictive value and immunological correlations of SECTM1 in the PRJEB23709 cohort (A) SECTM1 expression levels in tumors from patients with different responses. Data presented as mean ± SD. Significance was calculated with Student’s *t* test. ∗∗∗p < 0.001. (B) ORR in patients with low and high SECTM1 expression. Significance was calculated with Pearson’s χ^2^ test. ∗∗∗p < 0.001. (C) Correlations between SECTM1 expression and OS and PFS time. Significance was calculated with Pearson correlation test. (D and E) Prognostic values of SECTM1 in terms of OS and PFS. Median SECTM1 expression was used as the cut-off value. Significance was calculated with log rank test. (F) Cox regression analysis of prognosis-related factors in melanoma patients. (G) Heatmap showing correlations between SECTM1 and immunomodulators expression, including chemokines, receptors, MHCs, immunoinhibitors, and immunostimulators. Significance was calculated with Pearson correlation test.

We first discovered the predictive value of SECTM1 for immunotherapy in the PRJEB23709 cohort, and pre-treatment samples were included in our analysis (n = 73). The expression of SECTM1 was significantly enhanced in tumor samples with CR/PR responses compared with that with SD/PD responses (Figure 2A). We next divided the patients into two groups according to the median expression of SECTM1 and found that patients with high SECTM1 expression exhibited a higher objective response rate (ORR) compared with patients with low SECTM1 expression (73.68% vs. 34.29%, Figure 2B). Concerning survival time, SECTM1 expression was positively correlated with overall survival (OS) time and progression-free survival (PFS) time (Figure 2C), and patients with high SECTM1 expression showed better OS and PFS (Figures 2D and 2E). In addition, cox regression analysis suggested that SECTM1 was a prognosis-related factor in melanoma patients receiving immunotherapy (Figure 2F). We also compared the predictive values of SECTM1, PD-L1, and IFN-γ in the PRJEB23709 cohort. Although SECTM1 reached high discrimination in identifying therapeutic responses (AUC = 0.733), it was still slightly below PD-L1 (AUC = 0.787) and IFN-γ (AUC = 0.749) (Figure S1). In addition, we also combined SECTM1 and PD-L1 expression, and the combination reached high discrimination (AUC = 0.801) (Figure S1). Moreover, we checked the correlations between SECTM1 and immunomodulators expression, which were associated with features of tumor immune microenvironment (TIME) and the responses to immunotherapy, and the results showed that SECTM1 was positively correlated with most immunomodulators (Figure 2G). Overall, these results suggest SECTM1 is a potential biomarker of benefit to immunotherapy at least in melanoma.

### SECTM1 predicts the responses to immunotherapy in multiple cancer types

**Figure 3:**
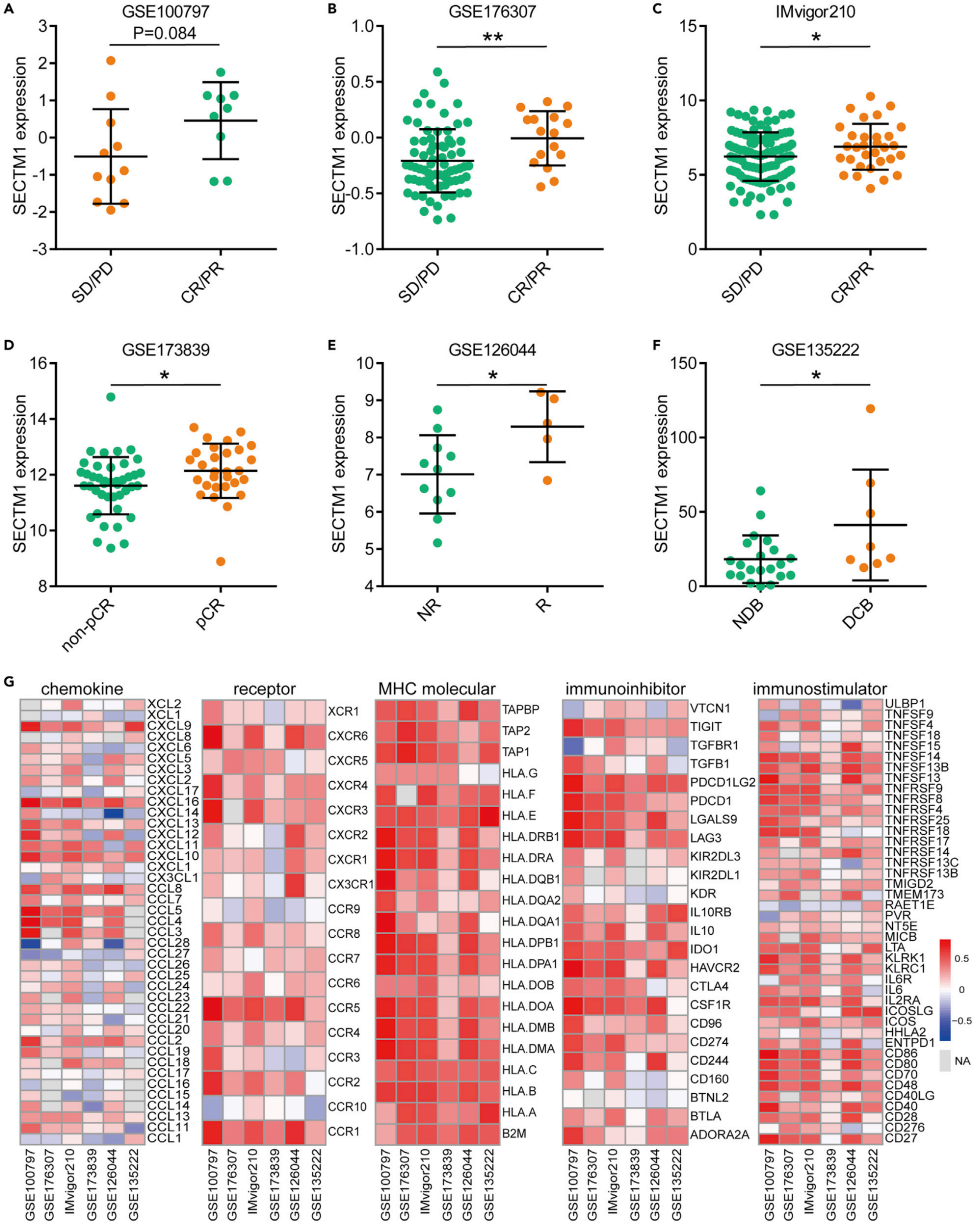
Predictive value and immunological correlations of SECTM1 in six cohorts (A–F) Expression levels of SECTM1 in tumors from patients with different responses, (A) the GSE100797 cohort, (B) the GSE176307 cohort, (C) the IMvigor210 cohort, (D) the GSE173839 cohort, (E) the GSE126044 cohort, and (F) the GSE135222 cohort. Data presented as mean ± SD. Significance for (A–E) was calculated with Student’s *t* test, and significance for (F) was calculated with Mann-Whitney test. ∗p < 0.05; ∗∗p < 0.01. (G) Heatmap showing correlations between SECTM1 and immunomodulators expression, including chemokines, receptors, MHCs, immunoinhibitors, and immunostimulators. Significance was calculated with Pearson correlation test.

To further verify the predictive value of SECTM1 for immunotherapy in human cancers, we obtained more public datasets. A total of six validated cohorts were used, namely the GSE100797 cohort (melanoma, n = 20, only samples collected before tumor-infiltrating lymphocytes [TILs] were included), the GSE176307 cohort (urothelial cancer, n = 88), the IMvigor210 cohort (urothelial cancer, n = 132, only samples collected pre-platinum were included), the GSE173839 cohort (breast cancer, n = 71), the GSE126044 cohort (lung cancer, n = 16), and the GSE135222 cohort (lung cancer, n = 27). Encouragingly, SECTM1 was remarkably increased in patients with well-therapeutic responses in these cohorts (Figures 3A–3F). In addition, SECTM1 showed higher discrimination in identifying therapeutic responses than PD-L1 in five cohorts, and the combination of SECTM1 and PD-L1 enhanced the predictive power (Figures S2A–S2F). Although IFN-γ showed satisfactory discrimination in several datasets, its stability did not seem to be good. In the GSE176307 and the GSE173839 cohorts, IFN-γ could not predict immunotherapeutic responses (Figures S2B and S2D). Also, SECTM1 was positively correlated with most immunomodulators in these cohorts (Figure 3G). Moreover, we investigated the association between SECTM1 and TIME features in the IMvigor210 and GSE176307 cohorts. SECTM1 expression was notably associated with PD-L1 expression in immune cells (IC) and tumor cells (TC) scores and was increased in inflamed tumors (Figures S3A–S3C). In addition, SECTM1 expression was positively correlated with neoantigen burden and tumor mutation burden (TMB) in the IMvigor210 and GSE176307 cohorts, respectively (Figures S3D–S3F). Collectively, these data reveal that SECTM1 could predict the responses to immunotherapy not only in melanoma but also in multiple cancer types.

### SECTM1 is associated with TIME across cancer types

**Figure 4:**
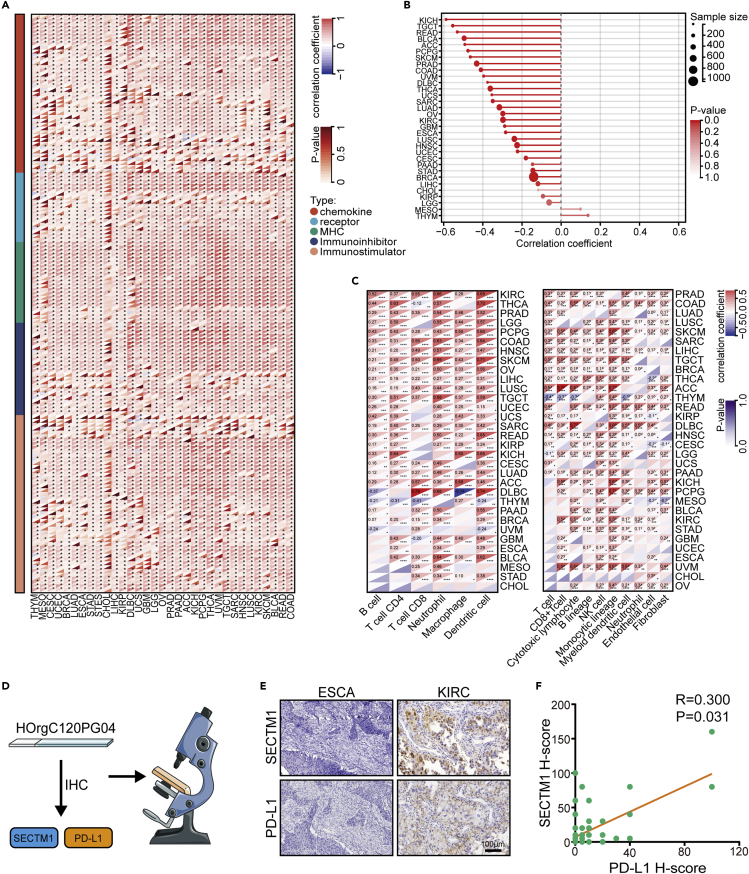
Pan-cancer analysis of immunological correlations of SECTM1 (A) Correlations between SECTM1 and immunomodulators expression in pan-cancer, including chemokine, receptor, MHC, immunoinhibitors, and immunostimulators. Significance was calculated with Pearson correlation test. (B) Correlation between SECTM1 and tumor purity in pan-cancer. Significance was calculated with Pearson correlation test. (C) Correlation between SECTM1 and TILs estimated by TIMER and MCP-counter algorithms in pan-cancer. Significance was calculated with Pearson correlation test. (D) Schematic protocol of validation using the pan-cancer TMA cohort. (E) Representative images revealing SECTM1 and PD-L1 expression in various tumor types. Magnification, 200X. (F) Correlation between SECTM1 and PD-L1 expression in the pan-cancer TMA cohort. Significance was calculated with Spearman correlation test.

Given that SECTM1 was positively correlated with most immunomodulators in the above cohorts, we next examined whether SECTM1 was associated with an inflamed TIME in pan-cancer using the TCGA dataset. We analyzed the correlations between SECTM1 and chemokine, receptor, major histocompatibility complex (MHC), immunoinhibitors, and immunostimulators. Except for CHOL, SECTM1 was positively correlated with the expression levels of these immunomodulators in almost all cancer types (Figure 4A). Moreover, SECTM1 was negatively correlated with tumor purity but positively correlated with TILs levels in most cancer types (Figures 4B and 4C). In addition, the correlations between SECTM1 and TMB as well as neoantigen burden were distinctive in different cancers, and SECTM1 showed high correlations with TMB and neoantigen burden in colorectal cancer (Figures S4A and S4B). We next collected a pan-cancer tissues microassay (TMA) cohort to validate the correlations between SECTM1 and TIME features (Figure 4D). The results showed that SECTM1 was highly correlated with PD-L1 expression in the cohort (Figures 4E and 4F). In addition, deficiency MMR was altofrequent and also significantly associated with immunotherapeutic responses in gastrointestinal cancer.^14^ Thus, we also assessed the associations between SECTM1 expression and MMR genes. In gastric cancer and colorectal cancer, SECTM1 was negatively correlated with MMR genes (Figures S5A and S5B). Taken together, these data uncover that SECTM1 is a pan-cancer classifier for immuno-hot tumors except for a few tumor types.

### SECTM1 is upregulated in immuno-hot tumors

**Figure 5:**
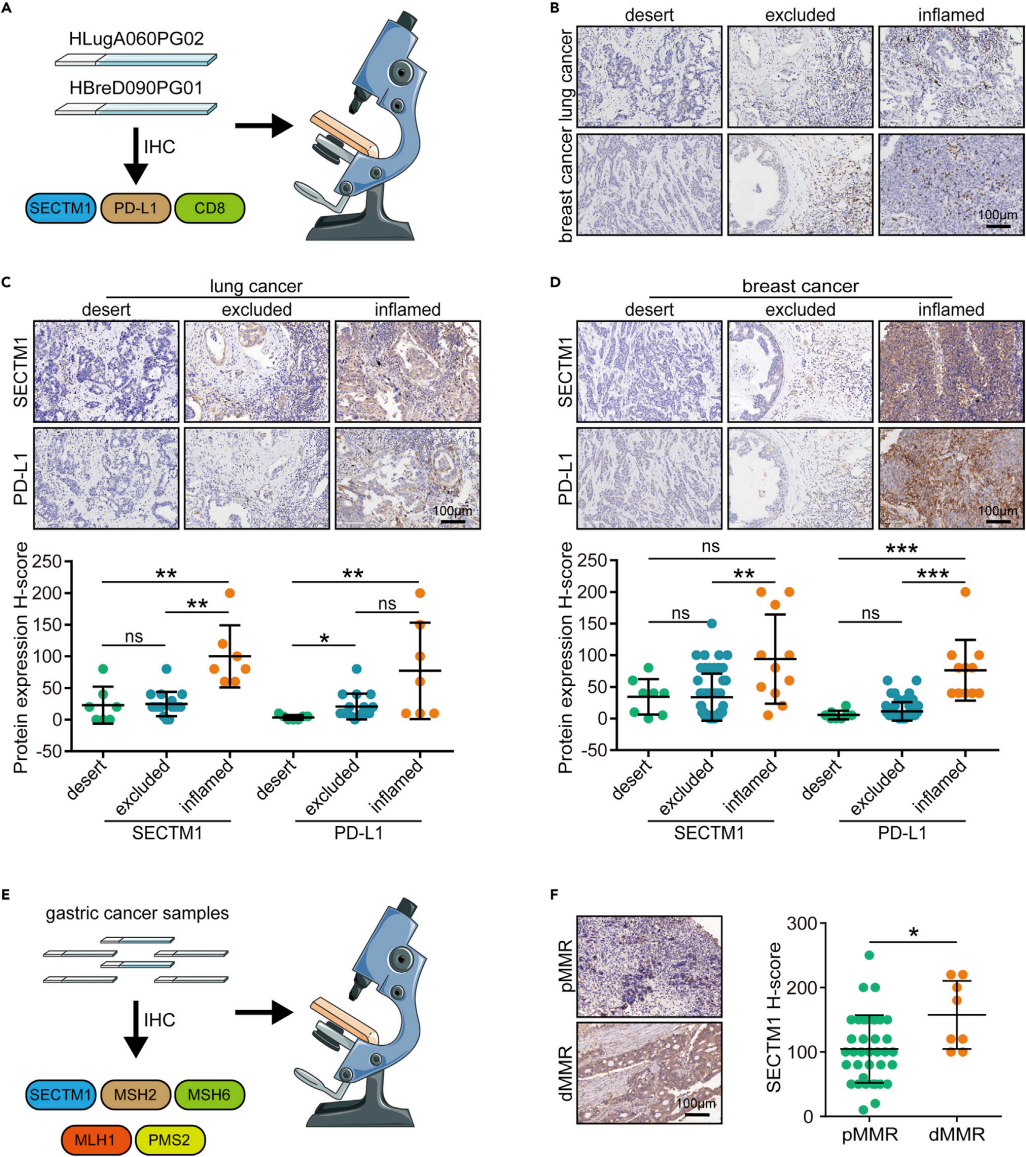
Correlation between SECTM1 expression and immuno-subtypes (A) Schematic protocol of validation on the TMA cohort. (B) Representative images revealing the distribution of CD8^+^ T cells in tumors with different immuno-subtypes. Magnification, 200X. (C) Representative images revealing SECTM1 and PD-L1 expression in tumors with different immuno-subtypes in lung cancer and semi-quantitative analysis of expression levels of SECTM1 and PD-L1. Magnification, 200×. Data are presented as mean ± SD. Significance was calculated with Kruskal-Wallis test with Tukey’s multiple-comparison test. ∗p < 0.05; ∗∗p < 0.01. (D) Representative images revealing SECTM1 and PD-L1 expression in tumors with different immuno-subtypes in breast cancer and semi-quantitative analysis of expression levels of SECTM1 and PD-L1. Magnification, 200×. Data are presented as mean ± SD. Significance was calculated with Kruskal-Wallis test with Dunn’s multiple-comparison test. ∗∗p < 0.01; ∗∗∗p < 0.001. (E) Schematic protocol of validation on the recruited gastric cancer cohort. (F) Representative images revealing SECTM1 expression in tumors with different MMR status in gastric cancer and semi-quantitative analysis of expression levels of SECTM1. Magnification, 200X. Significance was calculated with Mann-Whitney test. ∗p < 0.05.

Subsequently, we validated SECTM1 expression in tumors with different immuno-subtypes using the HLugA060PG02 and HBreD090PG01 cohorts (Figure 5A). We also compared SECTM1 expression between tumor and para-tumor tissues, and no significant difference was observed (Figures S6A and S6B). Figure 5B exhibited the discrimination of the inflamed, the excluded, and the deserted phenotypes based on the spatial distribution of CD8^+^ T cells. The results exhibited that SECTM1 and PD-L1 were associated with the immuno-subtypes, and these were the highest in immuno-inflamed tumors and the lowest in the immuno-desert tumors in lung and breast cancer (Figures 5C and 5D). In addition, SECTM1 was also positively correlated with PD-L1 in these cohorts (Figures S7A and S7B). We also validated the association between SECTM1 and MMR status in a recruited gastric cancer cohort (Figure 5E). The result showed that SECTM1 was notably overexpressed in tumors with deficiency of mismatch repair (dMMR) status compared with those with proficiency of mismatch repair (pMMR) status (Figures S8A, S8B, and 5F). IFN-γ is a cytokine secreted by CD8^+^ T cells and exerts anti-tumor effects, which is increased in immuno-hot tumors.^15^ SECTM1 was significantly upregulated by IFN-γ revealed by several public data (Figures S9A–S9D) and *in vitro* assay in NCI-H1299 and MDA-MB-231 cells. Given that the IFN-γ/STAT1 signaling is essential for both SECTM1 and PD-L1 expression,^12^^,^^16^ we used the STAT1 activation inhibitor fludarabine to inhibit STAT1 activation, and the IFN-γ-mediated SECTM1 and PD-L1 expression are both blocked (Figurse S10A and S10B). To sum up, SECTM1 is upregulated by IFN-γ and enriched in immuno-hot tumors.

### Predictive values of tumor-expressed and circulating SECTM1 in in-house cohorts

**Figure 6:**
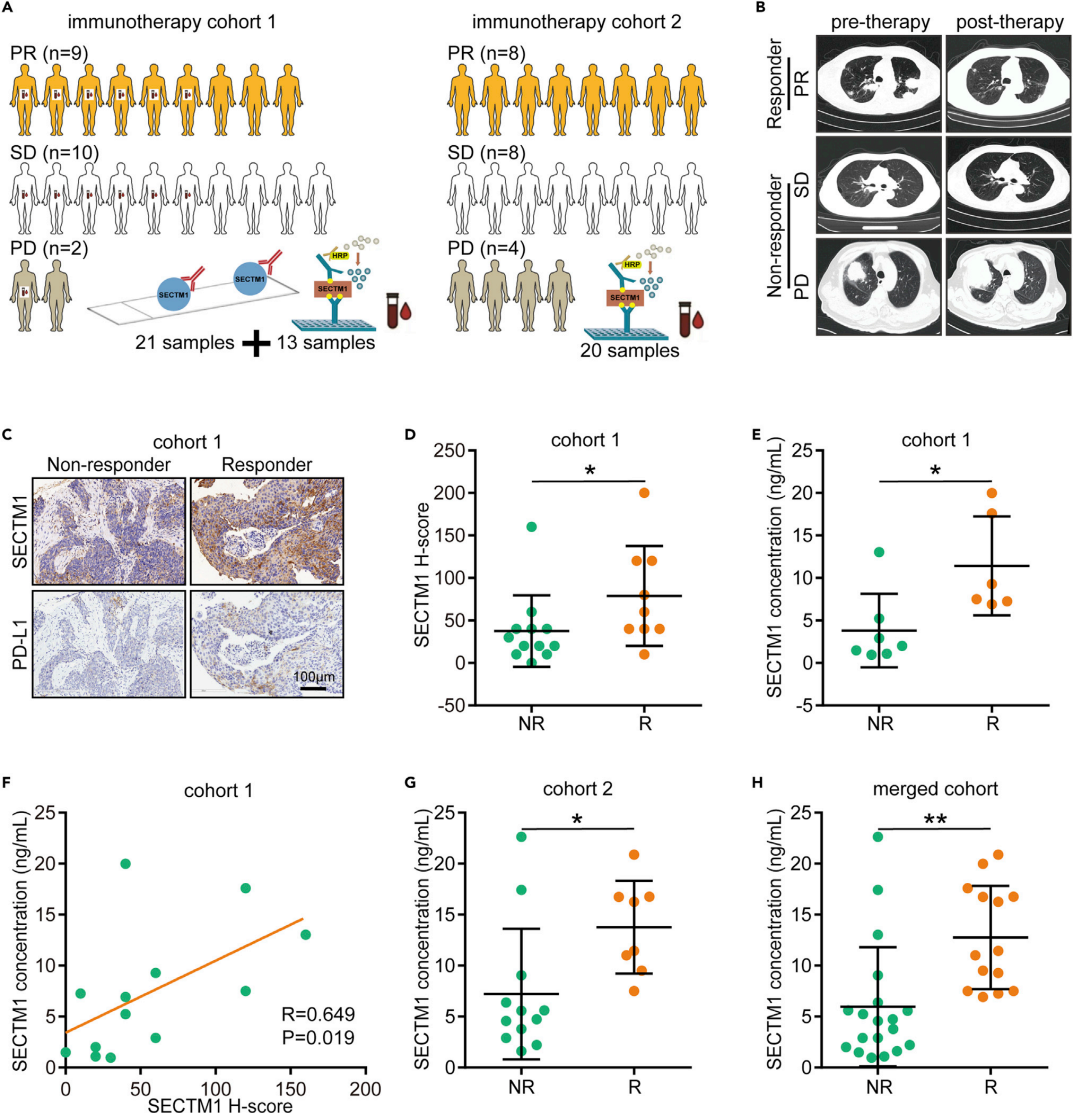
Validation of predictive value of SECTM1 for immunotherapy (A) Diagram of involved lung cancer cohorts in this research. (B) Representative CT images showing patients with different therapeutic responses. (C) Representative images uncovering SECTM1 and PD-L1 expression in tumors from patients with different responses. (D) Semi-quantitative analysis of expression of SECTM1 in tumors from patients with different responses in cohort 1. Significance was calculated with Mann-Whitney test. ∗p < 0.05. (E) Circulating SECTM1 levels in patients with different responses in cohort 1. Significance was calculated with Student’s *t* test. ∗p < 0.05. (F) Correlation between tumor-expressed and circulating SECTM1 in cohort 1. Significance was calculated with Spearman correlation test. (G and H) Circulating SECTM1 levels in patients with different responses in cohort 2 and merged cohort. Significance was calculated with Student’s *t* test. ∗p < 0.05; ∗∗p < 0.01.

We further validated the predictive value of SECTM1 for immunotherapy in in-house cohorts. Two independent lung cancer immunotherapy cohorts were included (Figures 6A and 6B). The therapeutic responses were evaluated according to the RECIST 1.1 criterion, and patients with PR response were deemed to be responders, and the others are considered to be nonresponders (Figure 6C). In cohort 1, SECTM1 and PD-L1 were highly expressed in tumor tissues from responders and positively correlated with each other (Figures 6C, 6D, and S11A), and the predictive value of SECTM1 (AUC = 0.769) was higher than that of PD-L1 (AUC = 0.718) (Figure S11B). Also, the combination of SECTM1 and PD-L1 showed higher predictive power (AUC = 0.806) (Figure S11B). Given that SECTM1 could be secreted to serum,^13^ we also collected the serum and examined the circulating SECTM1 levels. The results revealed that circulating SECTM1 in responders was higher than that in nonresponders (Figure 6E). In addition, circulating SECTM1 was positively correlated with tumor-expressed SECTM1 in cohort 1 (Figure 6F). Moreover, circulating SECTM1 in responders was higher than that in nonresponders in cohort 2 and the merged cohort (Figures 6G and 6H). Overall, tumor-expressed and circulating SECTM1 are both novel and promising biomarkers to predict the immunotherapeutic responses.

## Discussion

In recent years, increasing evidence has proved that TIME determines the responses to multiple anti-tumor therapies, especially immunotherapy.^17^ Immunomodulators and TILs are main components of the TIME, which are heterogeneous and dynamic.^18^ According to the features of the TIME, tumors could be divided into two subtypes, which included immuno-hot and immuno-cold tumors. Immuno-cold tumors are characterized by immunosuppressive TIME and the lack of TILs infiltration, and most solid immune-cold tumors are not responsive to immunotherapy. Contrarily, immuno-hot tumors are potential candidates which are responsive to immunotherapy.^18^^,^^19^ Thus, understanding the constitution of TIME within which immune cells function and identification of potential biomarkers related to the features of the TIME is significant for the discrimination of beneficiaries from immunotherapy in clinical practice.

Nowadays, the PD-L1 level has been established as a widely used biomarker to predict the therapeutic responses to ICIs in multiple solid cancer types since the first evidence supporting PD-L1 protein expression and the efficacy of anti-PD-1 therapy.^20^ PD-L1 expression is notably associated with the features of TIME, which is significantly enhanced by the IFN-γ signaling pathway and upregulated in immuno-hot tumors.^19^ However, a large group of patients with PD-L1-negative expression could benefit from immunotherapy, indicating that PD-L1 may not be an accurate biomarker of benefit to immunotherapy.^7^^,^^8^ Previous research indicated that deglycosylated PD-L1 could more effectively predict the immunotherapeutic responses,^21^^,^^22^ but the complex detection processes may limit its clinical application.

In the current research, we discovered that SECTM1 was upregulated in tumors from patients responsive to immunotherapy and associated with immunomodulators in the TIME utilizing a panel of public cohorts. In addition, we also conducted a pan-cancer analysis and found that SECTM1 was positively correlated with the inflamed TIME, and the positive correlation between SECTM1 and PD-L1 was validated in multiple in-house cohorts. More importantly, the predictive value of tumor-expressed SECTM1 for immunotherapy was also validated using in-house cohorts. SECTM1 is significantly associated with immune system processes and promotes the proliferation of T and NK cells.^12^ In soft tissue sarcoma, a signature consisting of SECTM1 and other four immune-related genes, IFIH1, CTSG, STC2, and BIRC5, could predict prognosis and the responses to ICIs^23^ These evidence further enhanced the correlation of SECTM1 with anti-tumor immunity. In addition, we also explored the regulation of SECTM1 on PD-L1, but the results showed that SECTM1 knockdown did not affect PD-L1 expression. However, IFN-γ/STAT1 signaling is essential for both SECTM1 and PD-L1 expression, which explains the co-expression pattern of SECTM1 with PD-L1 and the elevation of SECTM and PD-L1 in the inflamed tumors.

However, further studies on SECTM1 in tumors are lacking; thus, the functional role of SECTM1 is still unclear. Although the available evidence indicates that SECTM1 is an immunostimulator and activates multiple immune cells via CD7-dependent manner, SECTM1 still has a cancer-promoting effect in tumors. In melanoma, SECTM1 is produced by tumor cells and attracts human monocytes via CD7-mediated activation of the PI3K pathway, leading to cancer progression by attracting monocytes or macrophages.^13^ Thus, we speculate whether SECTM1 is an oncogene or a tumor suppressor dependent on the composition of immune cell types in the TIME, since multiple immune cells could express CD7, such as T cells, NK cells, and monocytes.^13^^,^^24^

As a minimally invasive strategy, the superiorities of liquid biopsy over tissue biopsy have been broadly manifested. Multiple biomarkers obtained from liquid biopsy, including soluble PD-L1, circulating immune cells, circulating noncoding RNA, peripheral blood cytokines, and circulating tumor DNA, could predict the responses to ICIs at baseline and monitor changes in the TIME during the treatment.^25^ Previous research indicated that SECTM1 could be detected in serum from melanoma patients but not normal donors,^13^ we also examined the predictive value of circulating SECTM1 and uncovered that circulating SECTM1 was positively correlated with tumor-expressed SECTM1 and could effectively predict the responses to ICIs. These data raised the possibility that SECTM1 could be used as serum biomarker.

In conclusion, our current study systemically analyzes the predictive value of SECTM1 for the responses to immunotherapy and its immunological correlations and identifies SECTM1 as a promising biomarker of benefit to immunotherapy in multiple cancer types in more than ten public and in-house cohorts. However, due to the limited number of cases in involved cohorts, further studies including large-scale patients are needed to establish its role as a biomarker of benefit to ICIs.

### Limitations of the study

Admittedly, the current research still has some limitations. First, as a less-focused immune-related molecule, the biological function of SECTM1 in cancer has not been well understood. The effect of SECTM1 on TIME and immunotherapy needs to be further explored. In addition, the predictive value of SECTM1 for immunotherapy was only validated in two small-scale in-house cohorts, which needs to be further validated in more large-scale cohorts with various cancer types. Moreover, it is undeniable that immuno-hot tumors are not always related to favorable prognosis and immunotherapeutic response.^26^ We revealed that SECTM1 could predict immunotherapeutic responses and the predictive power was based on immuno-hot features, and this implied that SECTM1 did not always work.

## STAR★Methods

### Key resources table

**Table:** 

REAGENT or RESOURCE	SOURCE	IDENTIFIER
**Antibodies**		

SECTM1 antibody	ProteinTech	Cat# 60281-1-Ig, RRID: AB_2881399
CD8 antibody	Abcarta	Cat# PA067, RRID: AB_2881399
PD-L1 antibody	GeneTech	Cat# GT2280, RRID: AB_2928127
MLH1 antibody	GeneTech	Cat# GT2304, RRID: AB_2928126
MSH2 antibody	GeneTech	Cat# GT2310, RRID: AB_2928128
MSH6 antibody	GeneTech	Cat# GT2195, RRID: AB_2928129
PSM2 antibody	GeneTech	Cat# GT2149, RRID: AB_2928130
PD-L1 antibody	ProteinTech	Cat# 66248-1-Ig, RRID: AB_2756526
Tubulin antibody	ProteinTech	Cat# 10094-1-AP, RRID: AB_2210695
STAT1 antibody	CST	Cat# 14994, RRID: AB_2737027
p-STAT1 antibody	CST	Cat# 9167, RRID: AB_561284

**Biological samples**		

Fetal bovine serum	Gibco	10091148
lung cancer TMA	Outdo	HLugA060PG02
breast cancer TMA	Outdo	HBreD090PG01
lung cancer samples	This study	NA
gastric cancer samples	This study	NA

**Chemicals, peptides, and recombinant proteins**		

Leibovitz’s L-15 medium	KeyGEN	KGM41300N-500
RPMI-1640 medium	KeyGEN	KGM31800N-500
IFN-γ	KeyGEN	KGH2016-10
Fludarabine	MCE	HY-B0069

**Critical commercial assays**		

SECTM1 ELISA kit	Cloud-Clone	SEM195hu

**Deposited data**		

PRJEB23709	TIDE	http://tide.dfci.harvard.edu/
GSE100797	TIDE	http://tide.dfci.harvard.edu/
IMvigor210	IMvigor210	http://research-pub.gene.com/IMvigor210CoreBiologies/
GSE176307	GEO	https://www.ncbi.nlm.nih.gov/geo/
GSE173839	GEO	https://www.ncbi.nlm.nih.gov/geo/
GSE126044	GEO	https://www.ncbi.nlm.nih.gov/geo/
GSE135222	GEO	https://www.ncbi.nlm.nih.gov/geo/
TCGA RNA-seq data	TCGA	https://xenabrowser.net/datapages/
TCGA mutation data	TCGA	https://portal.gdc.cancer.gov/
GSE199107	GEO	https://www.ncbi.nlm.nih.gov/geo/
GSE163067	GEO	https://www.ncbi.nlm.nih.gov/geo/
GSE85898	GEO	https://www.ncbi.nlm.nih.gov/geo/

**Experimental models: Cell lines**		

MDA-MB-231	KeyGEN	KG033, RRID: CVCL_0062
NCI-H1299	KeyGEN	KG307, RRID: CVCL_0060

**Software and algorithms**		

SPSS 26	IBM SPSS	https://www.ibm.com/docs/zh/spss-statistics/
Graphpad Prism 6.0	GraphPad	https://www.graphpad.com/
Sangerbox	Sangerbox	https://vip.sangerbox.com/login.html
R 4.0.2	R project	https://cran.r-project.org

### Resource availability

#### Lead contact

Further information and requests for resources and reagents should be directed to and will be fulfilled by the lead contact, Yongmei Yin (ymyin@njmu.edu.cn).

#### Materials availability

This study did not generate new unique reagents.

### Experimental model and subject details

#### Clinical cohorts

The paraffin-embedded lung cancer (Cat. HLugA060PG02) and breast cancer (Cat. HBreD090PG01) tissue microarrays (TMAs) and pan-cancer TMA (Cat. HOrgC120PG04) were purchased from Outdo BioTech (Shanghai, China). The HLugA060PG02 cohort contained 30 tumor and para-tumor samples, the HBreD090PG01 cohort contained 70 tumor samples and 20 para-tumor samples, and the pan-cancer HOrgC120PG04 cohort contained 11 kinds of cancers with 2–6 tumor samples and para-tumor or normal samples per type. Detailed clinic-pathological characteristics were obtained from Outdo BioTech. Ethical approval for the use of TMAs was granted by the Clinical Research Ethics Committee in Outdo Biotech (Shanghai, China).

A total of 45 paraffin-embedded gastric cancer samples were collected in The Affiliated Wuxi People’s Hospital of Nanjing Medical University from Jan. 2017 to Jun. 2022. The baseline characteristics could be found in Table S1. The mismatch repair (MMR) status of these samples were checked by using immunohistochemistry (IHC) staining of MSH2, MSH6, MLH1, and PMS2. Loss of expression of either protein was judged as dMMR or pMMR otherwise. In addition, a total of 41 lung cancer patients receiving anti-PD-1 immunotherapy monotherapy or a combination of chemotherapy were recruited by The Affiliated Wuxi People’s Hospital of Nanjing Medical University from Jan. 2019 to Feb. 2022. The baseline characteristics could be found in Table S2. The therapeutic responses were evaluated according to the RECIST 1.1 criterion, which was demarcated into CR, PR, SD, and PD. These patients were divided into two independent cohorts. Cohort 1 included 21 paraffin-embedded tissue samples and 13 serum samples from lung cancer patients, and cohort 2 included 20 serum samples from lung cancer patients before receiving therapy. Ethical approval for the collection of samples was granted by Clinical Research Ethics Committee, Nanjing Medical University.

#### Cell lines and cell treatment

MDA-MB-231 (RRID: CVCL_0062) and NCI-H1299 (RRID: CVCL_0060) cell lines were purchased from KeyGEN (Nanjing, China). MDA-MB-231 cells were maintained in the Leibovitz’s L-15 medium supplemented with 10% (v/v) fetal bovine serum (Cat. 10091148, Gibco, Waltham, UK) at 37°C with 5% CO_2_. NCI-H1299 cells were maintained in the RPMI-1640 medium supplemented with 10% (v/v) fetal bovine serum at 37°C with 5% CO_2_. All experiments were performed with mycoplasma-free cells and all cell lines have recently been authenticated using short tandem repeat profiling.

For subsequent assays, MDA-MB-231 and NCI-H1299 cells were starved overnight and stimulated with IFN-γ (Cat. KGH2016-10, KeyGEN, Nanjing, China) (20 ng/mL) for 48 h. For the STAT1 inhibition, IFN-γ-stimulated cells were further treated with the STAT1 activation inhibitor fludarabine (Cat. HY-B0069, MedChemExpress, Dalian, China) (5 μM) for 24 h. Then, the total protein of treated cells was harvested with lysis buffer and submitted for Western blotting analysis to check SECTM1 and PD-L1 protein levels.

### Method details

#### Public datasets acquisition

A panel of public datasets comprising RNA-sequencing data from patients receiving immunotherapy were downloaded from the Gene Expression Omnibus (GEO, http://www.ncbi.nlm.nih.gov/geo/) or the Tumor Immune Dysfunction and Exclusion (TIDE, http://tide.dfci.harvard.edu/) databases, including PRJEB23709,^27^ GSE100797,^28^ GSE176307,^29^ GSE173839,^30^ GSE126044,^31^ and GSE135222,^32^ datasets. The expression and clinical data of the IMvigor210 cohort^33^ were obtained from the corresponding website (http://research-pub.gene.com/IMvigor210CoreBiologies/). Among these datasets, samples obtained after immunotherapy or other therapies were excluded. The standardized TCGA pan-cancer dataset was downloaded from the UCSC (https://xenabrowser.net/) database. We further extracted the RNA-sequencing data in addition to TCGA-LAML, which was log_2_(x+0.001) transformed for further analysis. The abbreviations for TCGA cancer types are shown in Table S3. In addition, to analyze the effect of IFN-γ stimulation on SECTM1 expression in tumor cells, we downloaded GSE199107,^34^ GSE163067,^35^ and GSE85898,^36^ datasets. We summarized the public datasets used in our research in Table S4.

#### Analysis of tumor immune microenvironment features

For pan-cancer analysis, the Sangerbox, an interactive tool, was used.^37^ The features of the tumor immune microenvironment (TIME) were mainly reflected based on the expression levels of immunomodulators, including chemokine, MHC, receptor, immunoinhibitors, and immunostimulators. We also estimated the levels of tumor-infiltrating lymphocytes (TILs) using two independent algorithms, including TIMER^38^ and MCP-counter^39^ algorithms. In addition, the simple nucleotide variation data of the TCGA samples processed by the “MuTect2” software^40^ was downloaded from the Genomic Data Commons (GDC, https://portal.gdc.cancer.gov/) website. W used the R package “maftools” to calculate the tumor mutation burden (TMB) of each sample. Moreover, tumor purity and neoantigen mutation burden were obtained from a previous study.^41^ The correlations between SECTM1 and immunomodulators, TILs, TMB, tumor purity, and neoantigen mutation were evaluated.

#### Immunohistochemistry and semi-quantitative evaluation

IHC staining was conducted on the above TMAs and tissue slides. The primary antibodies used in the research were as follows: anti-SECTM1 (1:100 dilution, Cat. 60281-1-Ig, ProteinTech, Wuhan, China), anti-CD8 (Ready-to-use, Cat. PA067, Abcarta, Suzhou, China), anti-PD-L1 (Ready-to-use, Cat. GT2280, GeneTech, Shanghai, China), anti-MSH2 (Ready-to-use, Cat. GT2310, GeneTech, Shanghai, China), anti-MSH6 (Ready-to-use, Cat. GT2195, GeneTech, Shanghai, China), anti-MLH1 (Ready-to-use, Cat. GT2304, GeneTech, Shanghai, China), and anti-PMS2 (Ready-to-use, Cat. GT2149, GeneTech, Shanghai, China). Antibody staining was visualized with DAB and hematoxylin counterstain. All stained sections were independently evaluated by two independent pathologists. For semi-quantitative evaluation of SECTM1 and PD-L1 staining, the H-score criterion was used. In addition, tumors were demarcated into three phenotypes based on the spatial distribution of CD8^+^ T cells, including the inflamed, the excluded, and the deserted subtypes.^42^^,^^43^ The inflamed subtype is considered to be immuno-hot, and excluded and deserted subtypes are considered to be immuno-cold.^44^

#### Western blotting analysis

The total protein of treated cells was harvested and SDS-polyacrylamide gel electrophoresis and Western blotting analysis were conducted as standard protocols. The primary antibodies for SECTM1 (1:1000 dilution, Cat. 60281-1-Ig, ProteinTech, Wuhan, China), PD-L1 (1:1000 dilution, Cat. 66248-1-Ig, ProteinTech), STAT1 (1:1000 dilution, Cat. 14994, CST), p-STAT1 (1:1000 dilution, Cat. 9167, CST), and Tubulin (1:2000 dilution, Cat. 10094-1-AP, ProteinTech) were used. Protein expression levels were normalized to Tubulin for each sample.

#### Enzyme-linked immunosorbent assay

The enzyme-linked immunosorbent assay (ELISA) kit for SECTM1 (Cat. SEM195hu) was obtained from Cloud-Clone (Wuhan, China). The levels of plasma SECTM1 in patients from immunotherapy cohorts were examined by the ELISA test according to the manufacturer’s protocol.

### Quantification and statistical analysis

Statistical analysis and figure exhibition were performed using R language 4.0.2, Graphpad Prism 6.0, SPSS 26, and Sangerbox. Heatmap was generated by R package “pheatmap”. All data are presented as means ± SDs. The statistical difference of continuous variables between the two groups was evaluated by the Student t test or Mann-Whitney test according to the applicable conditions. The difference between multiple groups was analyzed by one-way ANOVA or Kruskal-Wallis test with multiple comparisons according to the applicable conditions. The chi-square test was used when the categorical variables were assessed. Pearson or Spearman correlation test was used to evaluate the correlation between two variables according to the applicable conditions. The predictive value of the combination of SECTM1 and PD-L1 was estimated by binary logistic regression. Receiver-operating characteristic (ROC) analysis was plotted to assess the specificity and sensitivity of the candidate indicator, and the area under the ROC curve (AUC) was generated for diagnostic biomarkers. Prognostic values of categorical variables were assessed by log-rank test and Cox regression analysis. For all analyses, p value <0.05 was deemed to be statistically significant and labeled with ∗p < 0.05; ∗∗p < 0.01; ∗∗∗p < 0.001.

### Additional resources

No additional resource was used in this study.

## Data Availability

This paper analyzes existing, publicly available data. These accession numbers for the datasets are listed in the key resources table. This paper does not report original code. Any additional information required to reanalyze the data reported in this paper is available from the lead contact upon request.
