# SECTM1 is upregulated in immuno-hot tumors and predicts immunotherapeutic efficacy in multiple cancers

**DOI:** 10.1016/j.isci.2023.108302

**Published:** 2023-10-25

**Authors:** Jie Mei, Ziyi Fu, Yun Cai, Chenghu Song, Jiaofeng Zhou, Yichao Zhu, Wenjun Mao, Junying Xu, Yongmei Yin

## Main text

(iScience *26*, 106027; February 17, 2023)

In the originally published version of this article, in Figure 3F, the FC and P values were mistakenly imported into the NDB group. The figure has been updated to reflect the correct values.

Additionally, in the Figure 5C legend, significance was calculated with Kruskal-Wallis test with Dunn’s multiple-comparison test, but this was mistakenly written as "Significance was calculated with Kruskal-Wallis test with Tukey’s multiple-comparison test."

These errors have now been corrected in the article online. The authors apologize for any confusion these errors may have caused.

**Figure:**
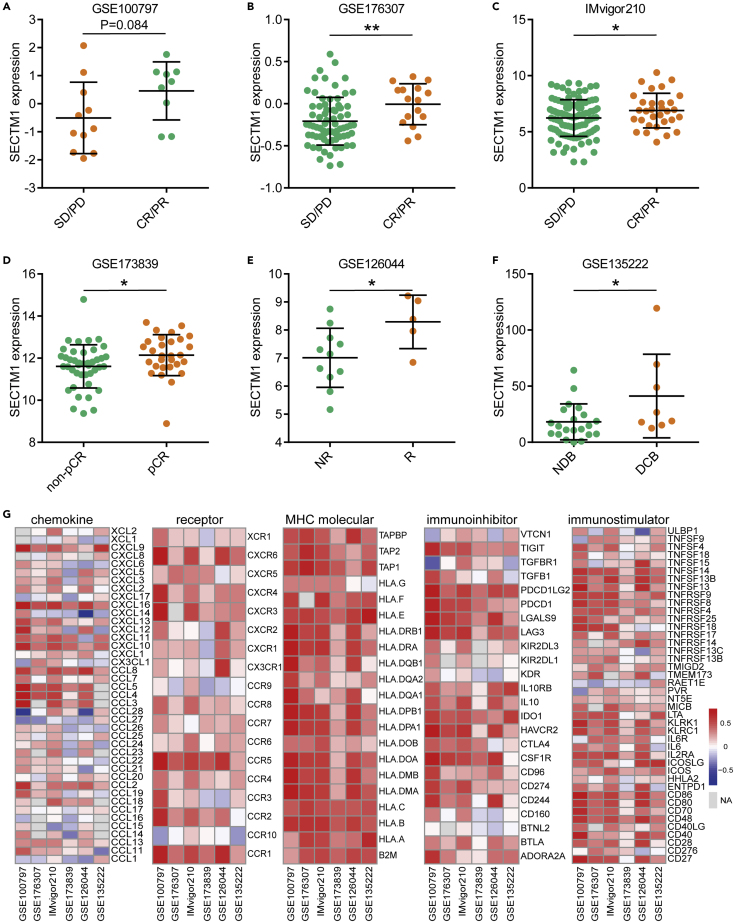
Figure 3. Predictive value and immunological correlations of SECTM1 in six cohorts (original)

**Figure:**
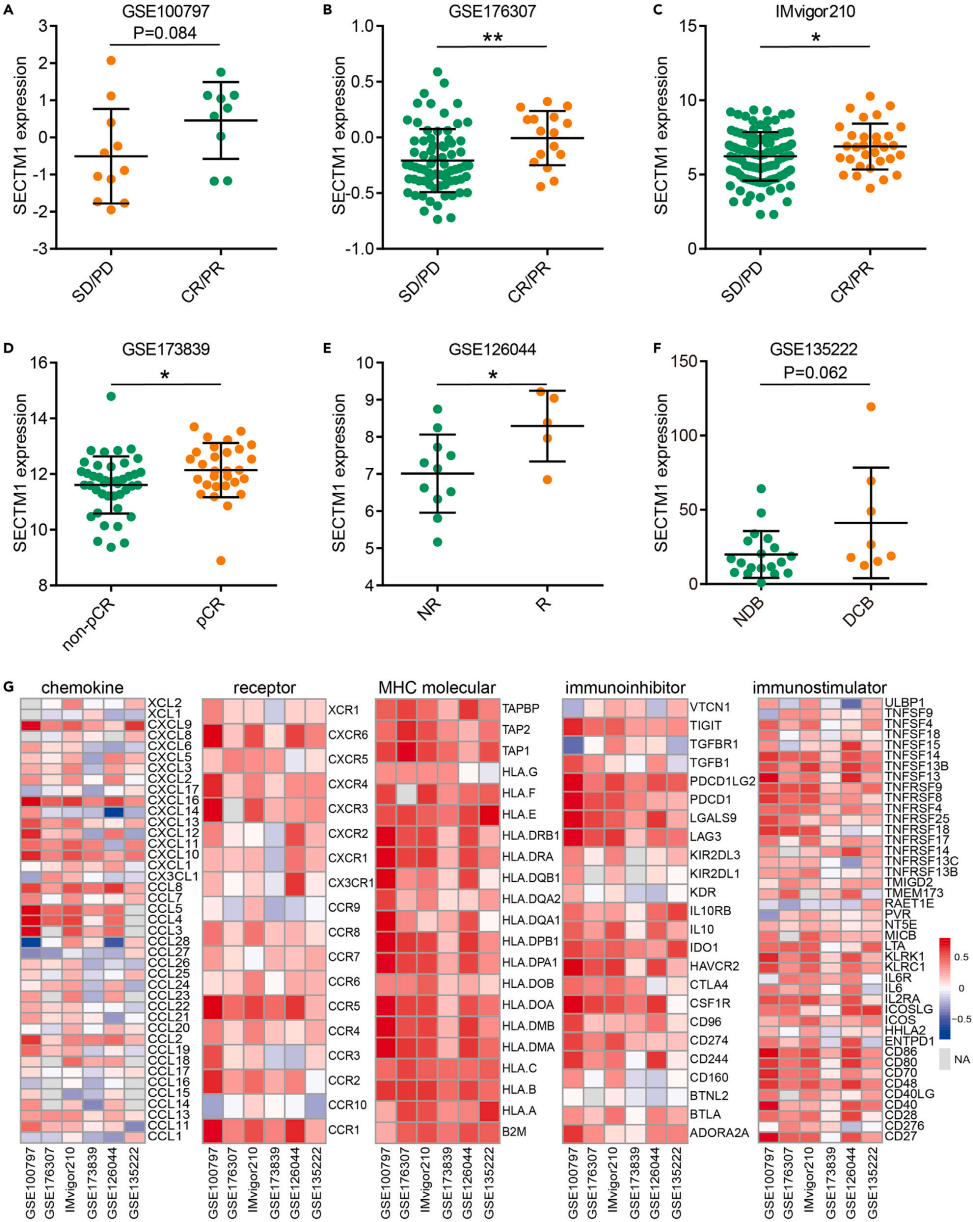
Figure 3. Predictive value and immunological correlations of SECTM1 in six cohorts (corrected)

