# Supplementary material for: SECTM1 is upregulated in immuno-hot tumors and predicts immunotherapeutic efficacy in multiple cancers
Source: iScience. 2023 Jan 23;26(2):106027. doi: 10.1016/j.isci.2023.106027 (PMC9932126; doi:10.1016/j.isci.2023.106027)
Supplement: Document S1 Figures S1–S11 and Tables S1–S4 [file mmc1.pdf]

iScience, Volume *26*

Supplemental information

SECTM1 is upregulated in immuno-hot tumors and predicts immunotherapeutic efficacy in multiple cancers

Jie Mei, Ziyi Fu, Yun Cai, Chenghu Song, Jiaofeng Zhou, Yichao Zhu, Wenjun Mao, Junying Xu, and Yongmei Yin

Supplementary figures

Figure S1. Comparison of predictive values of SECTM1, PD-L1, IFN-γ and the SECTM1/PD-L1 combination for immunotherapy in the PRJEB23709 cohort, related to Figure 2.

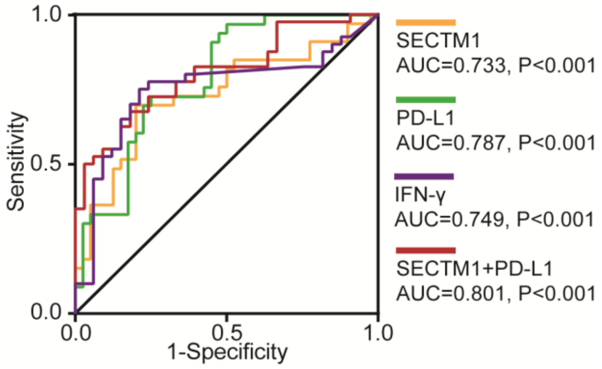

Figure S2. Comparison of predictive values of SECTM1, PD-L1, IFN-γ and the SECTM1/PD-L1 combination for immunotherapy in six cohorts, related to Figure 3.

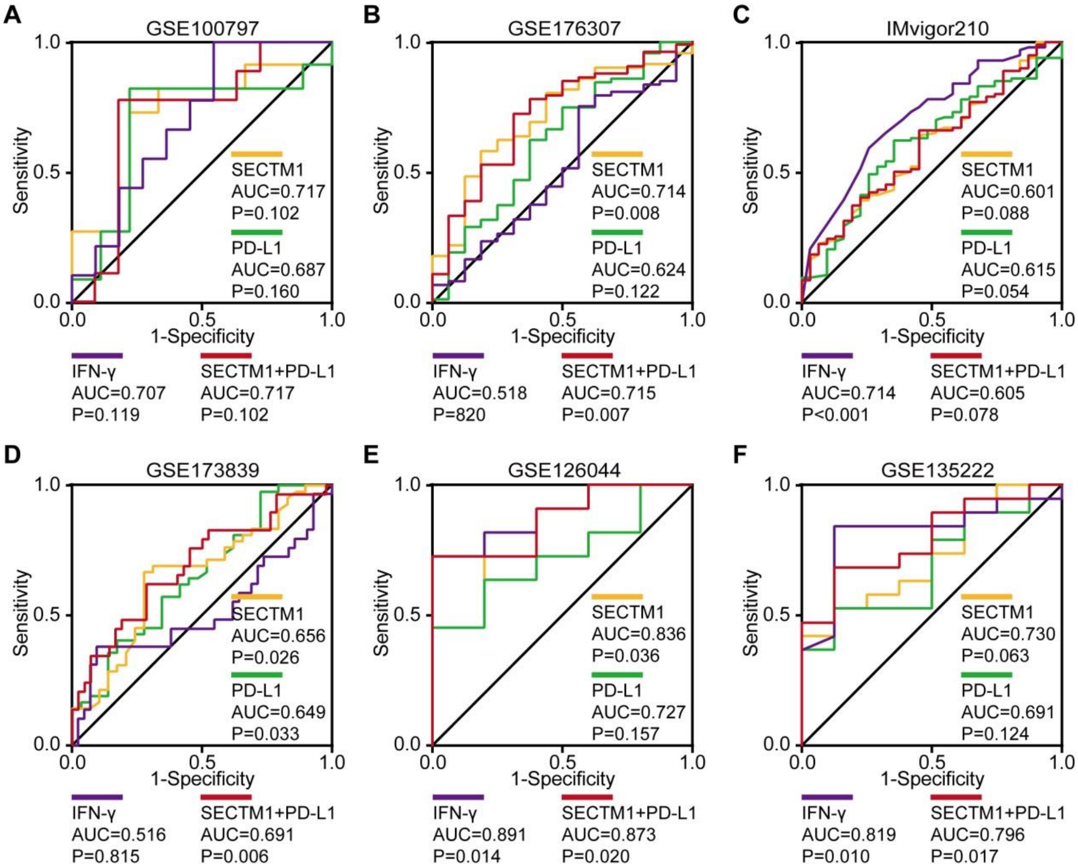

(A) The GSE100797 cohort. (B) The GSE176307 cohort. (C) The IMvigor210 cohort. (D) The GSE173839 cohort. (E) The GSE126044 cohort. (F) The GSE135222 cohort.

Figure S3. Associations between SECTM1 expression and features of TIME, related to Figure 3.

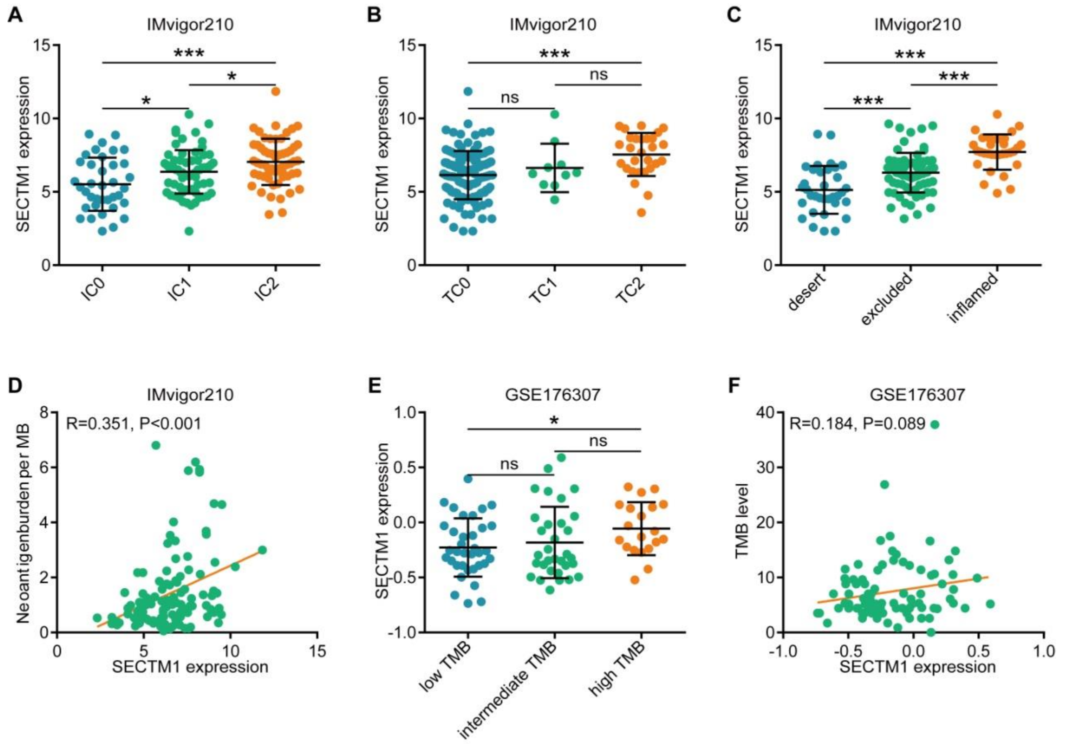

(A) Expression of SECTM1 in tumors with various PD-L1 IC score. Data are presented as mean ± SD. Significance was calculated with 1-way ANOVA with Tukey's multiple-comparison test. *P < 0.05; ***P < 0.001.

(B) Expression of SECTM1 in tumors with various PD-L1 TC score. Data are presented as mean ± SD. Significance was calculated with 1-way ANOVA with Tukey's multiple-comparison test. ***P < 0.001.

(C) Expression of SECTM1 in tumors with various immuno-subtypes. Data are presented as mean ± SD. Significance was calculated with 1-way ANOVA with Tukey's multiple-comparison test. ***P < 0.001.

(D) Correlation between SECTM1 expression and neoantigen burden. Significance was calculated with Pearson correlation test.

(E) Expression of SECTM1 in tumors with various TMB levels. Data are presented as mean ± SD. Significance was calculated with 1-way ANOVA with Tukey's multiple-comparison test. *P < 0.05.

(F) Correlation between SECTM1 expression and TMB levels. Significance was calculated with Pearson correlation test.

Figure S4. Correlations between SECTM1 expression and mutation burden, related to Figure 4.

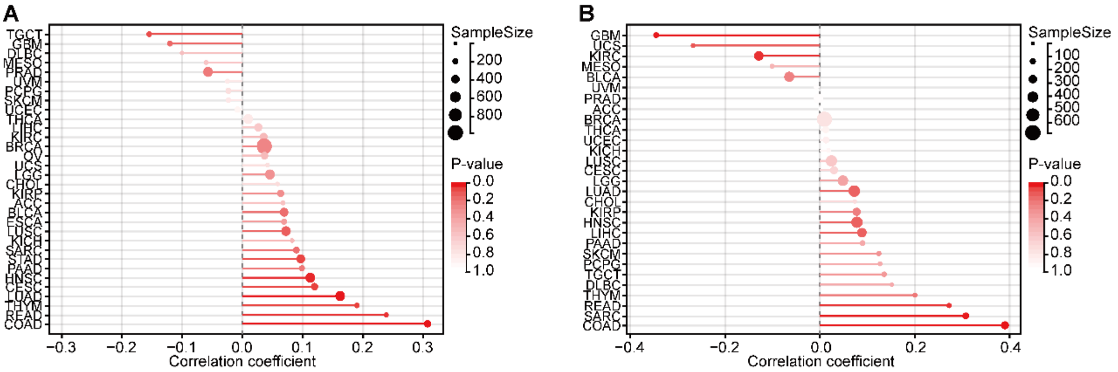

(A) Correlation between SECTM1 expression and TMB levels in pan-cancer. Significance was calculated with Pearson correlation test.

(B) Correlation between SECTM1 expression and neoantigen burden in pan-cancer. Significance was calculated with Pearson correlation test.

Figure S5. Correlations between SECTM1 and DNA mismatch repair genes, related to Figure 4.

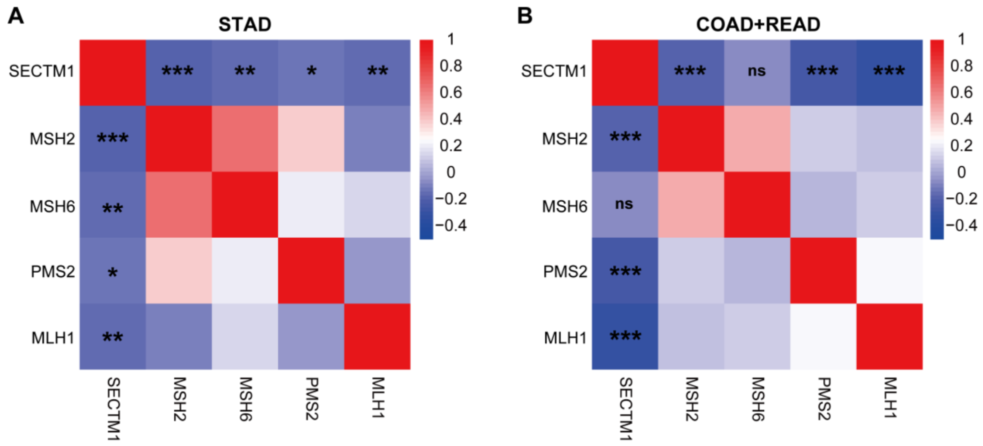

(A) SECTM1 was negatively correlated with DNA mismatch repair genes in gastric cancer. Significance was calculated with Pearson correlation test.*P < 0.05; **P < 0.01; ***P < 0.001.

(B) SECTM1 was negatively correlated with DNA mismatch repair genes in colorectal cancer. Significance was calculated with Pearson correlation test.***P < 0.001.

Figure S6. Comparison of SECTM1 expression in tumor and para-tumor tissues, related to Figure 5.

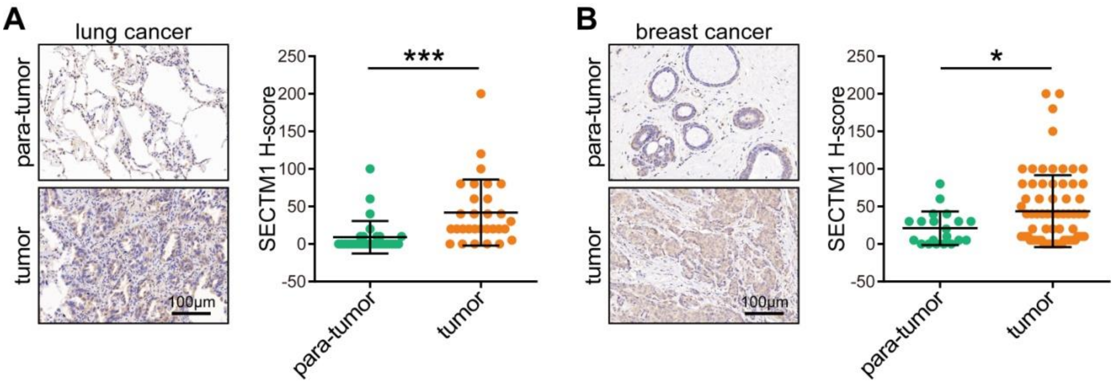

(A) Representative images revealing SECTM1 expression in tumor and para-tumor tissues in lung cancer and semi-quantitative analysis of expression levels of SECTM1. Magnification, 200×. Significance was calculated with Mann-Whitney test. ***P < 0.001.

(B) Representative images revealing SECTM1 expression in tumor and para-tumor tissues in breast cancer and semi-quantitative analysis of expression levels of SECTM1. Magnification, 200×. Significance was calculated with Mann-Whitney test. *P < 0.05.

Figure S7. Correlations between SECTM1 and PD-L1 expression, related to Figure 5.

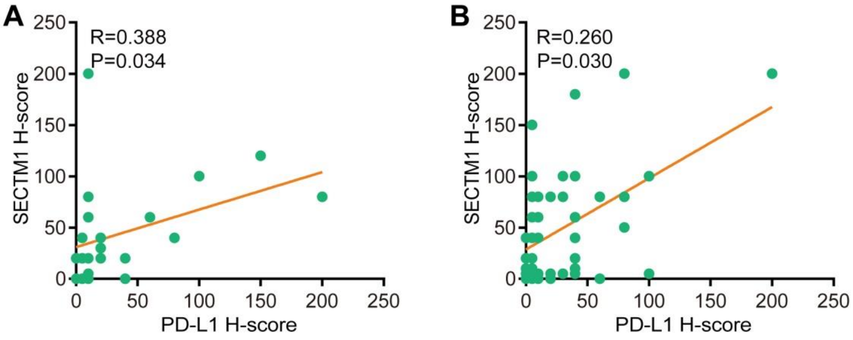

(A) Correlation between SECTM1 and PD-L1 expression in the HLugA060PG02 cohort. Significance was calculated with Spearman correlation test.

(B) Correlation between SECTM1 and PD-L1 expression in the HBreD090PG01 cohort. Significance was calculated with Spearman correlation test.

Figure S8. Expression of MMR proteins in gastric cancer, related to Figure 5.

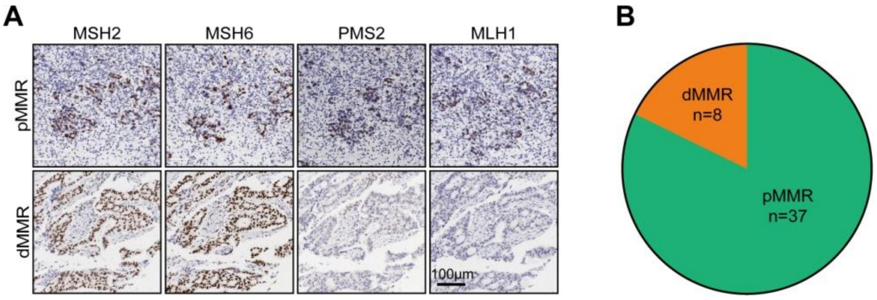

(A) Representative images revealing MMR proteins expression in gastric cancer and corresponding proportions. Magnification, 200×.

Figure S9. Transcriptional change of SECTM1 expression under the stimulation of IFN-γ, related to Figure 5.

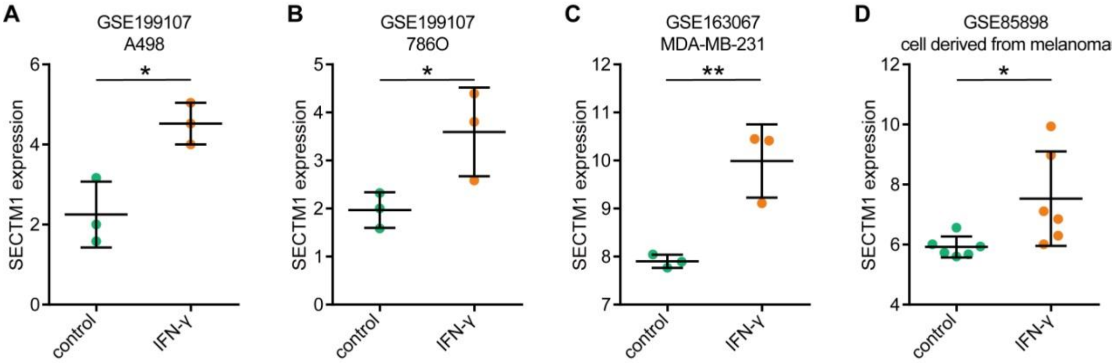

(A) Data of A498 cells from the GSE199107 dataset. Significance was calculated with Student's t test. *P < 0.05.

(B) Data of 786O cells from the GSE199107 dataset. Significance was calculated with Student's t test. *P < 0.05.

(C) Data of MDA-MB-231 cells from the GSE163067 dataset. Significance was calculated with Student's t test. **P < 0.01.

(D) Data of melanoma cells from the GSE85898 dataset. Significance was calculated with Mann-Whitney test. *P < 0.05.

Figure S10. Regulation of IFN-γ/STAT1 signaling on the expression of SECTM1 and PD-L1, related to Figure 5.

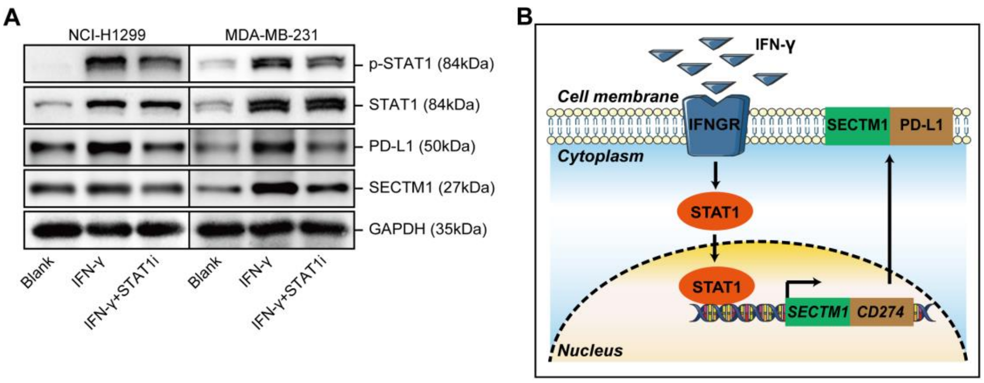

(A) Expression levels of SECTM1 and PD-L1 after IFN-γ stimulation or STAT1 inhibition.

(B) Schematic diagram of the mechanism underlying SECTM1 and PD-L1 are common downstream genes of the IFN-γ/STAT1 signaling.

Figure S11. Comparison of predictive values of SECTM1 and PD-L1 for immunotherapy in the in-house cohorts, related to Figure 6.

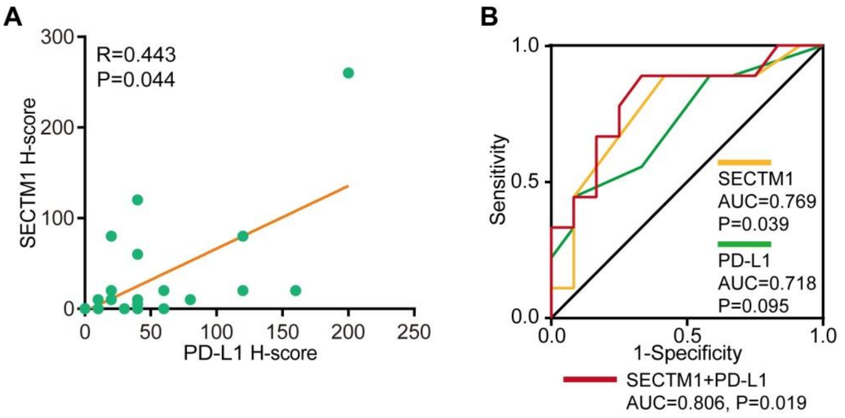

(A) Correlation between tumor-expressed SECTM1 and PD-L1 expression in cohort 1. Significance was calculated with Spearman correlation test.

(B) Comparison of predictive values of tumor-expressed SECTM1 and PD-L1 in cohort 1.

Supplementary tables

Table S1. Baseline features of recruited gastric cancer cohorts, related to Figure 5.

clinic-pathological parameters	number	proportion (%)
gender		
male	35	77.78%
female	10	22.22%
age		
<70	31	68.89%
≥70	14	31.11%
clinical stage		
stage 1-2	9	20.00%
stage 3-4	36	80.00%
differentiation		
medium	11	24.44%
poor	34	75.56%
MMR status		
dMMR	8	17.78%
pMMR	37	82.22%

Table S2. Baseline features of combined lung cancer immunotherapy cohorts, related to Figure 6.

clinic-pathological parameters	number	proportion (%)
gender		
male	30	73.17%
female	11	26.83%
age		
<70	23	56.10%
≥70	18	43.90%
clinical stage		
stage 3	11	26.83%
stage 4	30	73.17%
differentiation		
medium	19	46.34%
poor	22	53.66%
pathological type		
squamous cell carcinoma	16	39.02%
adenocarcinoma	16	39.02%
others	9	21.95%
therapeutic response		
PR	17	41.46%
SD	18	43.90%
PD	6	14.63%
sample type		
histological section	8	19.51%
serum	20	48.78%
both	13	31.71%

Table S3. Table of abbreviations in the TCGA database, related to Figure 1.

abbreviation	full name
ACC	adrenocortical carcinoma
BLCA	bladder urothelial carcinoma
BRCA	breast invasive carcinoma
CESC	cervical squamous cell carcinoma and endocervical adenocarcinoma
CHOL	cholangiocarcinoma
COAD	colon adenocarcinoma
DLBC	lymphoid neoplasm diffuse large B-cell lymphoma
ESCA	esophageal carcinoma
GBM	glioblastoma multiforme
HNSC	head and neck squamous cell carcinoma
KICH	kidney chromophobe carcinoma
KIRC	kidney renal clear cell carcinoma
KIRP	kidney renal papillary cell carcinoma
LGG	brain lower grade glioma
LIHC	liver hepatocellular carcinoma
LUAD	lung adenocarcinoma
LUSC	lung squamous cell carcinoma
MESO	mesothelioma
OV	ovarian serous cystadenocarcinoma
PAAD	pancreatic adenocarcinoma
PCPG	pheochromocytoma and paraganglioma
PRAD	prostate adenocarcinoma
READ	rectum adenocarcinoma
SARC	sarcoma
SKCM	skin cutaneous melanoma
STAD	stomach adenocarcinoma
TGCT	testicular germ cell tumor
THCA	thyroid carcinoma
THYM	tthymoma
UCEC	uterine corpus endometrial carcinoma
UCS	uterine carcinosarcoma
UVM	uveal melanoma

Table S4. Public cohorts used in the current research, related to Figure 1.

cohort	cancer type	application
PRJEB23709	melanoma	discovery cohort
GSE100797	melanoma	validated cohort
GSE176307	urothelium cancer	validated cohort
IMvigor210	urothelium cancer	validated cohort
GSE173839	breast cancer	validated cohort
GSE126044	lung cancer	validated cohort
GSE135222	lung cancer	validated cohort
TCGA	pan-cancer	pan-cancer analysis
GSE199107	A498, 786O	SECTM1 expression analysis
GSE163067	MDA-MB-231	SECTM1 expression analysis
GSE85898	melanoma cells	SECTM1 expression analysis
